# Supplementary material for: Triamidoamine thorium-arsenic complexes with parent arsenide, arsinidiide and arsenido structural motifs
Source: Nat Commun. 2017 Mar 9;8:14769. doi: 10.1038/ncomms14769 (PMC5347141; doi:10.1038/ncomms14769)
Supplement: Supplementary Information — Supplementary Figures, Supplementary Tables, Supplementary Methods and Supplementary References [file ncomms14769-s1.pdf]

Supplementary Figures

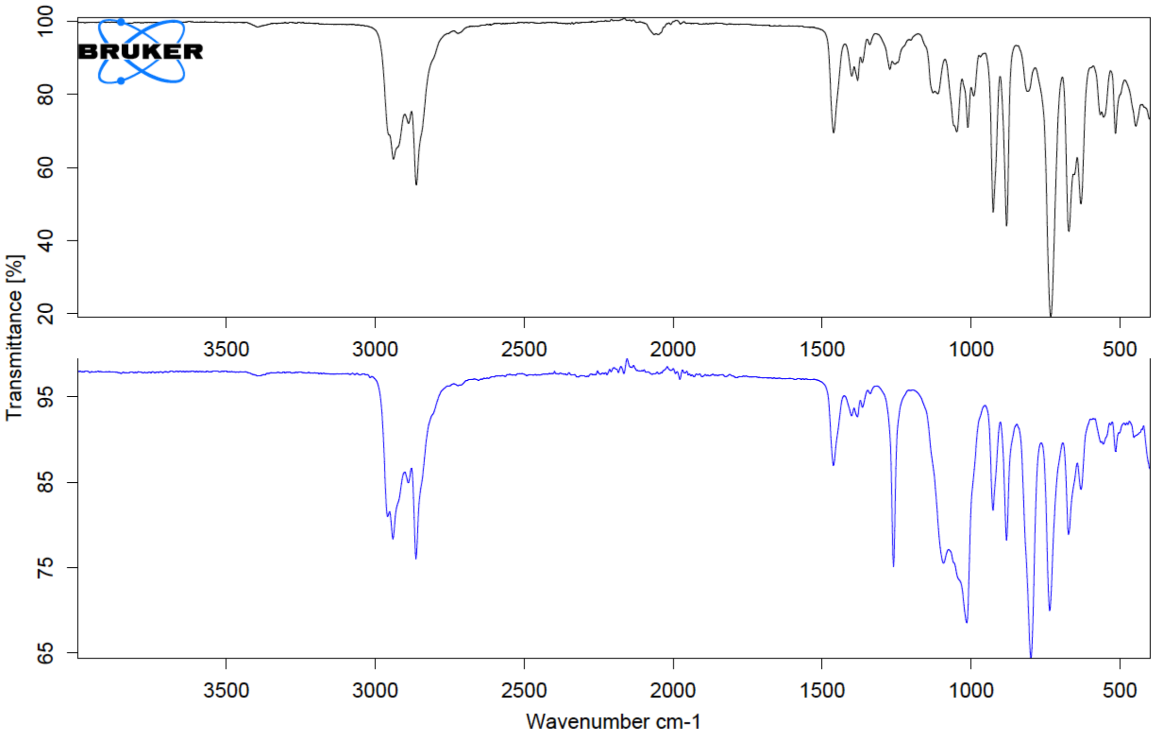

Supplementary Figure 1. ATR-IR spectra of 2 (top) and 2D (bottom).

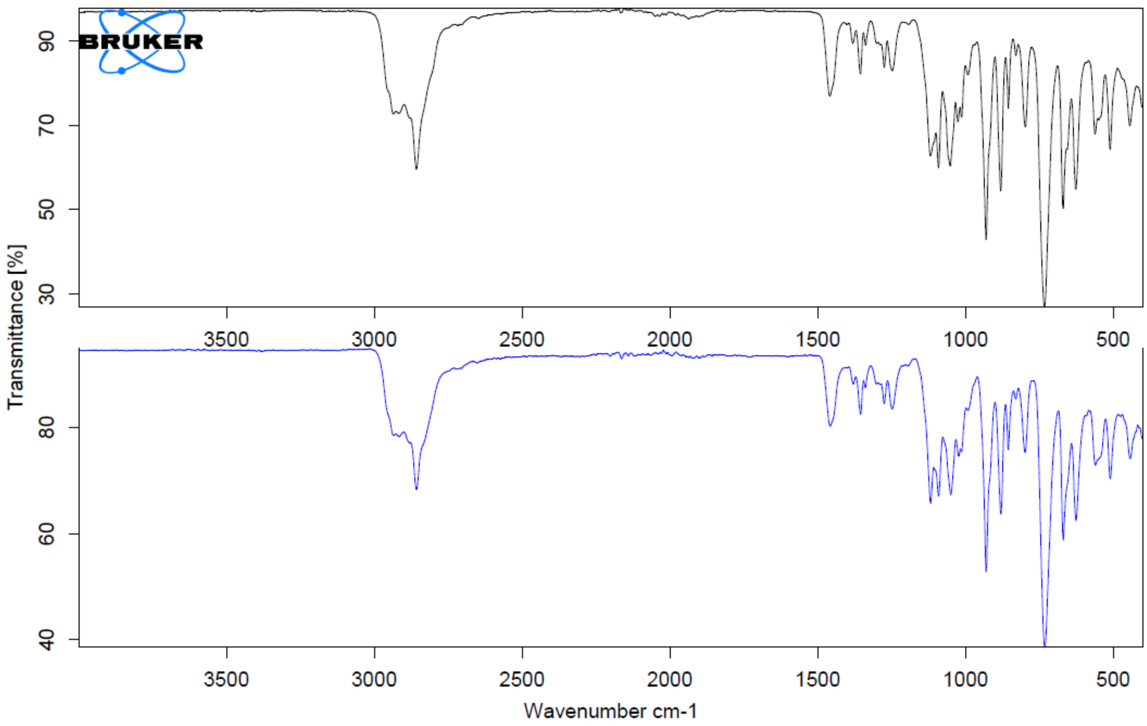

Supplementary Figure 2. ATR-IR spectra of 3 (top) and 3D (bottom).

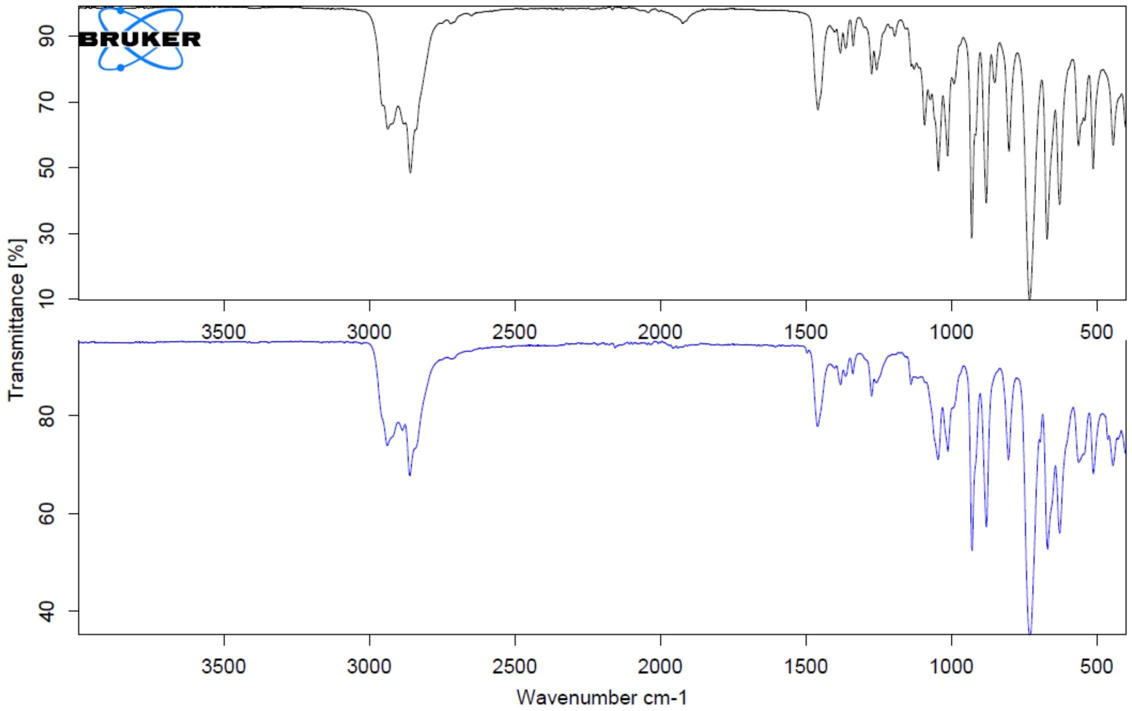

Supplementary Figure 3. ATR-IR spectra of 5 (top) and 5D (bottom).

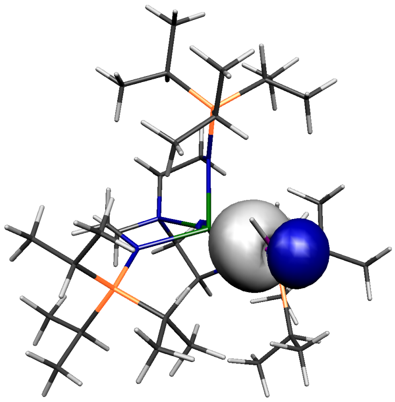

Supplementary Figure 4. NBO representation of the Th-As σ-bond in 2.

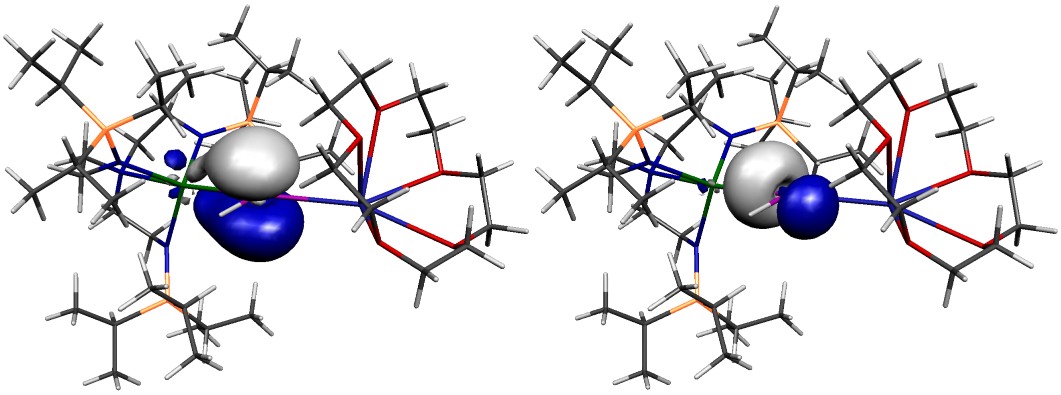

Supplementary Figure 5. NBO representation of the Th-As π-bond (left), NBO representation of the Th-As σ-bond (right) in 3.

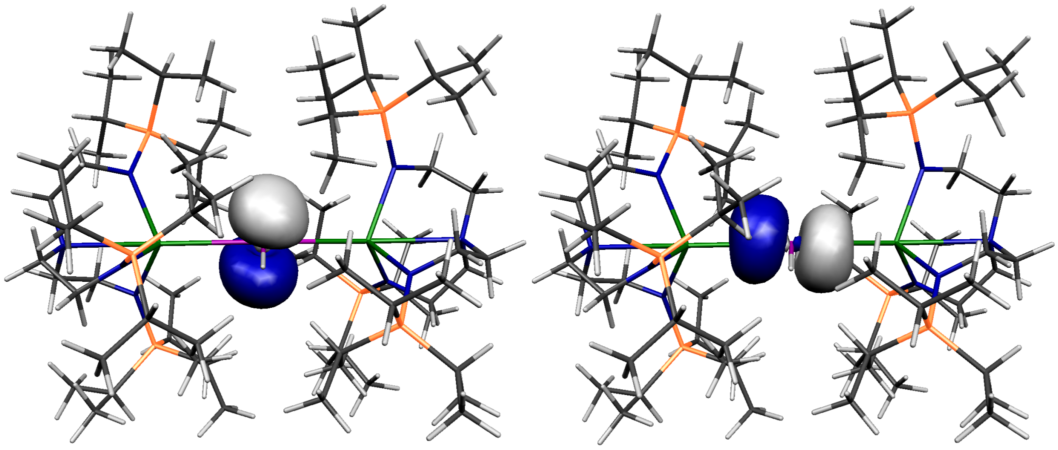

Supplementary Figure 6. NBO representation of the Th-As π-bonding interaction (left), NBO representation of the Th-As σ-bonding interaction (right) in 5.

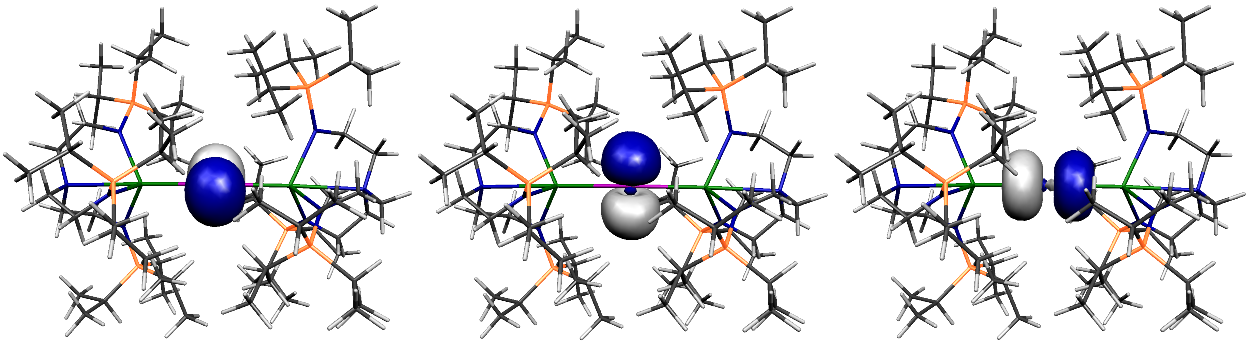

Supplementary Figure 7. Frontier orbitals of 6^-. Left to right: NBO representation of one of the Th-As π-bonding interactions, NBO representation of the other Th-As π-bonding interaction, NBO representation of the Th-As σ-bonding interaction in 6.

Supplementary Tables

Supplementary Table 1. Final Coordinates and Single Point Energy of 2 After Geometry Optimisation

1.C	-0.868866	-3.214145	-4.443910
2.C	-3.329886	-0.331626	-4.663219
3.C	-3.047601	-4.212185	-3.700364
4.C	-1.832346	-3.370449	-3.248794
5.C	-3.765734	-0.882372	-3.289098
6.C	0.030904	4.179027	-2.793837
7.C	-0.205217	-0.223924	-2.914857
8.C	1.245215	-0.680474	-2.767157
9.C	-4.500234	0.219337	-2.502795
10.C	0.631041	6.192911	-1.391165
11.C	2.363047	1.187232	-1.602772
12.C	0.695921	4.653448	-1.486042
13.C	-4.592560	-2.909623	-0.633055
14.C	-2.533343	2.746407	-0.785267
15.C	2.814100	-1.132076	-0.917977
16.C	-3.153366	-2.353367	-0.597864
17.C	2.170247	1.934629	-0.284163
18.C	-2.493278	4.956327	0.416547
19.C	2.135143	-2.145861	0.001598
20.C	-1.809225	3.578833	0.285013
21.C	-2.239750	-3.333572	0.161621
22.C	0.729284	4.711098	1.621194
23.C	2.264644	4.735447	1.760023
24.C	4.677887	-3.013664	2.890489
25.C	4.546719	-0.552706	2.368777
26.C	1.680638	-4.746913	2.255680
27.C	3.830890	-1.735130	3.053411
28.C	0.076008	4.288891	2.950332
29.C	1.315951	-3.623792	3.248233
30.C	1.100863	-0.611736	3.946106
31.C	1.556718	0.846723	3.760182
32.C	-0.200119	-3.665105	3.522272
33.C	1.282256	-1.011828	5.425972
34.H	-1.305636	-2.608232	-5.252483
35.H	-0.639988	-4.202708	-4.878118
36.H	-4.197270	0.074190	-5.211959
37.H	-2.870386	-1.098565	-5.303042
38.H	-2.717298	-5.184440	-4.104064
39.H	0.089580	-2.757107	-4.166108
40.H	-3.610263	-3.705552	-4.500435
41.H	-2.608007	0.492732	-4.555277
42.H	-0.567039	-0.553649	-3.901235
43.H	-4.496181	-1.692606	-3.482047
44.H	-3.752698	-4.418243	-2.884291
45.H	0.557932	4.584029	-3.674599

46.H	1.868975	-0.355304	-3.622807
47.H	-5.381532	0.577545	-3.061828
48.H	-1.292872	-3.960910	-2.483678
49.H	-1.012851	4.522923	-2.858192
50.H	1.258095	-1.778226	-2.738588
51.H	0.019258	3.083037	-2.888156
52.H	-0.232496	0.880628	-2.953219
53.H	-3.846885	1.086492	-2.338746
54.H	1.039244	6.654958	-2.306668
55.H	1.804326	1.716228	-2.385830
56.H	-4.851068	-0.117269	-1.516996
57.H	3.426873	1.155240	-1.908607
58.H	-2.540218	3.256228	-1.759290
59.H	-5.285088	-2.264639	-1.190663
60.H	3.383283	-1.629536	-1.726677
61.H	-0.403924	6.553344	-1.282238
62.H	1.769836	4.394608	-1.563356
63.H	-4.627712	-3.907646	-1.095209
64.H	-2.083455	1.754826	-0.973379
65.H	-2.428545	5.525379	-0.523949
66.H	1.208865	6.584905	-0.542020
67.H	2.662516	2.915997	-0.373656
68.H	-3.580448	2.564112	-0.498690
69.H	1.463399	-2.788009	-0.598832
70.H	3.522937	-0.548285	-0.318547
71.H	-2.192716	-4.309238	-0.345626
72.H	-4.989060	-3.014849	0.389766
73.H	-3.201923	-1.420024	0.002085
74.H	-3.563894	4.840650	0.653708
75.H	2.724436	1.405355	0.511857
76.H	2.912982	-2.825877	0.388020
77.H	-2.046577	5.574454	1.207753
78.H	-2.610364	-3.508192	1.183405
79.H	2.767500	5.078300	0.842683
80.H	4.745196	-3.334309	1.838415
81.H	-1.197256	-2.981621	0.252117
82.H	-1.947852	3.052832	1.250553
83.H	0.404911	5.748364	1.412919
84.H	4.762303	-0.776913	1.313113
85.H	1.156035	-4.610766	1.298050
86.H	4.281347	-3.857002	3.472158
87.H	-3.375124	0.819550	1.667984
88.H	5.711692	-2.836178	3.234421
89.H	2.573884	5.412337	2.574371
90.H	2.658947	3.737226	2.003907
91.H	2.756704	-4.802420	2.043146
92.H	3.965622	0.378223	2.396680
93.H	1.373196	-5.728673	2.654028
94.H	-1.013609	4.432796	2.943296
95.H	1.456109	1.210880	2.725012
96.H	0.265057	3.230356	3.175842
97.H	0.014681	-0.649899	3.732752

98.H	5.518023	-0.352174	2.851883
99.H	-0.771714	-3.413802	2.617661
100.H	-1.868746	0.924891	3.252109
101.H	0.479444	4.878807	3.791208
102.H	3.825881	-1.510954	4.139232
103.H	-0.515809	-2.965383	4.307123
104.H	0.967926	1.521864	4.400441
105.H	1.839430	-3.835691	4.201152
106.H	2.611155	0.975156	4.046176
107.H	-0.514385	-4.674506	3.834247
108.H	0.741689	-0.310808	6.082980
109.H	0.908280	-2.020900	5.644444
110.H	2.342300	-0.979251	5.724516
111.As	-2.539291	-0.205947	2.454032
112.N	-1.036674	-0.737439	-1.796889
113.N	1.814975	-0.189700	-1.487202
114.N	0.725986	2.043455	0.051549
115.N	1.390945	-1.452944	1.085868
116.Si	-2.393330	-1.784015	-2.273220
117.Si	0.097253	3.705088	0.100042
118.Si	1.914586	-1.862852	2.734578
119.Th	-0.272811	-0.043313	0.297770

Energy: −635.81207014 eV

Supplementary Table 2. Final Coordinates and Single Point Energy of 3 After Geometry Optimisation

1.C	-0.399645	3.848488	-6.410433
2.C	-0.352847	0.139977	-5.455851
3.C	-0.595733	3.933713	-4.881707
4.C	2.861345	3.706792	-4.593296
5.C	-2.089134	4.138232	-4.560449
6.C	2.058609	2.390203	-4.621850
7.C	-0.528088	0.782133	-4.064828
8.C	2.888818	1.248657	-4.008879
9.C	-2.007330	0.705747	-3.645101
10.C	0.854554	-7.102933	-2.294981
11.C	2.024856	-7.552771	-1.432425
12.C	0.706456	4.262896	-1.798371
13.C	-5.967198	1.776336	-1.426464
14.C	-4.886446	-0.503938	-1.431636
15.C	-0.412393	5.067068	-1.134060
16.C	-1.050562	-6.572126	-0.915126
17.C	4.143315	-6.788621	-0.584744
18.C	-5.408706	0.641270	-0.544437
19.C	-3.108596	3.500979	-0.418297
20.C	3.448564	1.264511	-0.188924
21.C	-1.493783	-5.514022	0.074363
22.C	-2.322953	4.572499	0.340467

23.C	4.001304	-6.411261	0.883580
24.C	3.071950	0.651792	1.171235
25.C	-0.051017	4.801601	1.283615
26.C	-3.415139	-0.318043	1.626634
27.C	-6.563495	1.973847	2.289054
28.C	4.311211	-0.030312	1.784682
29.C	3.667267	4.356458	1.795113
30.C	-5.121243	2.423259	1.971124
31.C	-0.726772	-4.378912	2.048780
32.C	4.013122	-4.471549	2.320516
33.C	-4.503506	-1.268347	2.166846
34.C	0.112670	3.639944	2.265036
35.C	-2.363226	-0.055959	2.717058
36.C	3.519254	3.230302	2.837381
37.C	2.755975	-4.722481	3.143018
38.C	0.378425	-4.451484	3.080579
39.C	-4.377761	2.764520	3.277967
40.C	1.346583	-0.558498	3.751558
41.C	1.830128	0.881318	4.013252
42.C	3.470767	3.837640	4.254453
43.C	0.824346	1.595275	4.939916
44.H	-0.849339	4.723434	-6.912353
45.H	-0.884457	2.953084	-6.830506
46.H	0.660591	3.814887	-6.699147
47.H	-0.909246	0.690029	-6.231552
48.H	0.699368	0.098447	-5.771071
49.H	1.884004	2.139131	-5.686213
50.H	-0.737070	-0.894558	-5.455109
51.H	3.814412	3.600474	-5.140514
52.H	-2.459139	5.090957	-4.977883
53.H	2.314746	4.545796	-5.050147
54.H	-2.705744	3.336355	-4.994032
55.H	-0.070753	4.851320	-4.549222
56.H	3.860793	1.143151	-4.522848
57.H	-2.665578	1.196184	-4.377598
58.H	2.374492	0.278541	-4.064686
59.H	3.115667	3.997189	-3.562061
60.H	-2.287822	4.147904	-3.479385
61.H	-2.330965	-0.343585	-3.559539
62.H	1.242478	-6.675917	-3.231896
63.H	0.035963	0.156745	-3.341348
64.H	0.230942	-7.976356	-2.551641
65.H	3.095850	1.439862	-2.946758
66.H	1.052492	4.841625	-2.672134
67.H	-2.218377	1.185096	-2.674121
68.H	2.490684	-8.449370	-1.886701
69.H	-6.767807	1.404648	-2.089431
70.H	-5.656289	-0.823090	-2.156465
71.H	-5.182071	2.196717	-2.073232
72.H	-4.004482	-0.193003	-2.008690
73.H	-1.261328	5.111438	-1.827980
74.H	-1.882658	-6.819052	-1.599426

75.H	4.969197	-6.209694	-1.022243
76.H	-2.892940	3.604110	-1.498339
77.H	-6.390300	2.603734	-0.836369
78.H	1.575760	4.232589	-1.114270
79.H	1.670079	-7.821965	-0.420301
80.H	4.392596	-7.861660	-0.663104
81.H	-0.097070	6.109059	-0.922780
82.H	-4.596471	-1.388506	-0.846339
83.H	-1.196023	-1.045316	-1.245452
84.H	3.805683	0.486129	-0.881697
85.H	-0.769454	-7.484802	-0.359081
86.H	2.604281	1.764722	-0.688846
87.H	-4.180273	3.744019	-0.322621
88.H	-1.600129	-4.532672	-0.425721
89.H	-6.253545	0.241076	0.048680
90.H	4.252540	2.009986	-0.088248
91.H	-2.643123	5.594945	0.053158
92.H	-2.479387	-5.798995	0.489711
93.H	4.873396	-6.808241	1.439999
94.H	-7.176249	1.862908	1.382707
95.H	3.672485	3.984300	0.761936
96.H	0.945681	5.079070	0.916794
97.H	-2.882156	-0.845807	0.809853
98.H	4.707998	-0.804377	1.104794
99.H	3.087574	-6.861254	1.311560
100.H	2.317968	-0.134547	0.971088
101.H	-5.212875	3.369889	1.404076
102.H	-2.520754	4.447737	1.413651
103.H	-0.717210	-3.398198	1.526760
104.H	-5.256870	-1.523903	1.407574
105.H	-0.489662	5.691373	1.779410
106.H	4.603878	4.919507	1.953046
107.H	5.127440	0.690926	1.952306
108.H	2.843136	5.081394	1.878601
109.H	-6.585568	1.009913	2.822358
110.H	-7.067848	2.712482	2.937072
111.H	-1.700763	-4.500688	2.558095
112.H	4.165638	-3.390637	2.204869
113.H	-1.895668	-1.000367	3.037063
114.H	-4.056875	-2.214970	2.519070
115.H	-1.539458	0.589293	2.374441
116.H	4.882572	-4.889810	2.861693
117.H	-0.878904	3.383201	2.684566
118.H	4.097670	-0.510115	2.750891
119.H	4.452879	2.633775	2.794313
120.H	-3.326615	3.038888	3.104648
121.H	-5.035702	-0.827564	3.025180
122.H	0.697279	4.016514	3.122870
123.H	-4.862808	3.606042	3.802770
124.H	0.391948	-5.455707	3.543459
125.H	2.103294	-1.174409	3.245937
126.H	2.612277	-5.802020	3.343191

127.H	0.451177	-0.575097	3.112861
128.H	-2.799633	0.420391	3.606332
129.H	2.883449	-4.217893	4.121096
130.H	0.200066	-3.699363	3.869208
131.H	-4.381403	1.909350	3.971613
132.H	4.350471	4.481593	4.430860
133.H	2.580812	4.470792	4.401135
134.H	2.797176	0.821247	4.551026
135.H	-0.164600	1.662866	4.463210
136.H	1.088455	-1.059174	4.702815
137.H	3.464886	3.069586	5.041155
138.H	1.136110	2.614879	5.205781
139.H	0.693982	1.034886	5.882560
140.As	0.106941	-1.158043	-0.408275
141.K	1.583936	-3.985169	-0.416631
142.N	0.255501	2.895885	-2.151317
143.N	-0.866826	4.399995	0.112861
144.N	-2.774667	2.139848	0.064955
145.N	0.742864	2.469828	1.609716
146.O	0.068552	-6.076908	-1.673129
147.O	2.983131	-6.485779	-1.376760
148.O	3.965862	-4.982623	0.978717
149.O	-0.508351	-5.450913	1.111447
150.O	1.637216	-4.203299	2.432785
151.Si	0.278707	2.515235	-3.874702
152.Si	-4.126657	1.250976	0.772492
153.Si	2.202968	1.865943	2.385662
154.Th	-0.467820	1.667983	-0.254905

Energy: −826.61823899 eV

Supplementary Table 3. Final Coordinates and Single Point Energy of 5 After Geometry Optimisation

1.C	-3.610243	-3.916416	-5.979073
2.C	0.164345	-3.736576	-6.026202
3.C	-2.608916	3.914829	-5.371191
4.C	-0.535283	-2.489149	-5.449228
5.C	0.492793	-1.388321	-5.144557
6.C	-3.246974	-0.637093	-4.599019
7.C	-2.950245	-4.190603	-4.611083
8.C	-4.741665	-0.665771	-4.288256
9.C	-4.134726	5.361930	-3.977160
10.C	-2.766123	4.648420	-4.024651
11.C	3.765733	0.144033	-3.885104
12.C	-4.004191	-4.690668	-3.604864
13.C	-0.206450	1.854326	-3.560846
14.C	7.275007	-0.916099	-3.066042
15.C	-5.250729	1.537109	-3.305190
16.C	0.520141	4.230177	-3.189273

17.C	-0.397787	3.099409	-2.679346
18.C	-0.359222	-4.970458	-2.597717
19.C	4.672458	-2.797561	-2.205123
20.C	3.231821	-3.335142	-2.226998
21.C	3.538587	0.086777	-2.361095
22.C	-6.065222	-0.514550	-2.208935
23.C	-4.639092	2.369551	-2.182801
24.C	-0.822371	-3.508838	-2.423413
25.C	6.659183	-0.477949	-1.719372
26.C	5.617582	-3.845509	-1.581361
27.C	6.918590	1.025218	-1.496297
28.C	3.417303	1.512442	-1.799959
29.C	-1.827277	6.264990	-1.183325
30.C	-5.450316	-1.516143	-1.233615
31.C	-2.309368	4.822999	-0.918973
32.C	-1.643904	-3.352888	-1.126298
33.C	-3.666558	4.867743	-0.188663
34.C	3.448830	4.619371	-0.076564
35.C	1.603678	5.989348	0.933782
36.C	5.396937	-1.650183	1.134071
37.C	-6.986783	1.335530	1.222877
38.C	1.651990	-3.622748	1.281118
39.C	2.808484	5.077568	1.245677
40.C	-5.981783	-3.556965	1.589246
41.C	-6.790350	-0.158193	1.547238
42.C	-3.465801	1.673931	1.696252
43.C	6.102116	-0.636261	2.030674
44.C	4.794343	2.320129	2.065392
45.C	-3.554210	-3.199325	2.173064
46.C	-4.953539	-2.562798	2.168969
47.C	-3.655698	0.261757	2.275319
48.C	0.777056	-3.360792	2.519202
49.C	-7.453153	-0.481899	2.904327
50.C	0.590048	3.162057	2.619780
51.C	-0.072305	-4.609678	2.829550
52.C	5.427258	1.455549	3.152056
53.C	-0.359007	4.271114	3.122203
54.C	0.315284	1.891020	3.450878
55.C	3.934556	-4.607202	3.805278
56.C	-3.891385	0.349079	3.796593
57.C	4.881856	-0.721399	4.166160
58.C	4.405482	4.994655	4.326684
59.C	2.974223	4.424786	4.228185
60.C	3.415105	-0.618694	4.572301
61.C	2.984603	-3.941414	4.820265
62.C	-0.742532	-1.441509	4.951158
63.C	2.681673	3.562830	5.471032
64.C	0.405743	-2.345639	5.428294
65.C	2.347710	-5.027738	5.715063
66.C	0.966541	-1.814545	6.764057
67.H	0.711897	-3.481852	-6.949972
68.H	-2.883398	-3.607366	-6.744436

69.H	-4.114735	-4.823396	-6.354994
70.H	-2.871434	4.580650	-6.211893
71.H	-1.195033	-2.098352	-6.248100
72.H	-0.539075	-4.545133	-6.275055
73.H	1.123164	-1.176542	-6.024516
74.H	-4.379529	-3.130683	-5.915741
75.H	-3.079515	-1.124019	-5.574517
76.H	-1.581034	3.567356	-5.542364
77.H	-3.269185	3.035265	-5.438414
78.H	0.904385	-4.144180	-5.320174
79.H	-5.350722	-0.268406	-5.123111
80.H	-4.211340	6.102329	-4.792482
81.H	-2.247734	-5.032478	-4.771380
82.H	-2.934211	0.413540	-4.742620
83.H	0.005912	-0.452747	-4.846381
84.H	1.167237	-1.681959	-4.325730
85.H	-0.609900	1.984847	-4.574049
86.H	2.953302	0.696306	-4.385010
87.H	3.807098	-0.855246	-4.340001
88.H	-5.039558	-1.709280	-4.122035
89.H	-4.756854	1.808952	-4.247589
90.H	-4.969712	4.656965	-4.114116
91.H	-2.002367	5.452201	-4.010832
92.H	4.706277	0.661586	-4.130511
93.H	6.769244	-0.441151	-3.920752
94.H	0.227561	4.578276	-4.192290
95.H	-4.580772	-5.532972	-4.025809
96.H	0.860930	1.605625	-3.663099
97.H	8.338462	-0.625489	-3.118117
98.H	-6.334757	1.728831	-3.422779
99.H	7.225787	-2.003788	-3.217717
100.H	4.989344	-2.661871	-3.257358
101.H	-4.304130	5.898126	-3.032444
102.H	-4.730607	-3.903349	-3.346551
103.H	-1.207759	-5.659900	-2.727685
104.H	1.563514	3.881450	-3.260791
105.H	-0.671525	0.941354	-3.152656
106.H	3.187659	-4.327300	-2.706693
107.H	-6.839538	-0.981842	-2.847357
108.H	-3.554602	-5.042029	-2.665657
109.H	2.549473	-2.673432	-2.778124
110.H	6.499491	1.627111	-2.317374
111.H	0.303294	-5.097828	-3.464624
112.H	0.518739	5.102041	-2.522893
113.H	5.589519	-4.786801	-2.156612
114.H	-4.916394	3.423974	-2.333772
115.H	2.552239	2.036378	-2.232564
116.H	2.565939	-0.414035	-2.186279
117.H	-2.511587	6.801633	-1.859207
118.H	6.665829	-3.512308	-1.552940
119.H	4.310034	2.114679	-2.016304
120.H	0.083448	-2.888374	-2.295859

121.H	-0.824897	6.308958	-1.629712
122.H	8.000929	1.238925	-1.461605
123.H	-4.967994	-2.326103	-1.810327
124.H	-6.548771	0.280303	-1.626585
125.H	-0.058222	2.824831	-1.661977
126.H	0.197534	-5.305291	-1.706774
127.H	2.829587	-3.444107	-1.209937
128.H	-5.118220	2.079571	-1.229598
129.H	7.217939	-1.020307	-0.931648
130.H	-2.721446	-3.524999	-1.254213
131.H	6.476251	1.397247	-0.560765
132.H	5.317590	-4.092462	-0.551709
133.H	-4.442517	5.361958	-0.794249
134.H	-6.270637	-2.004728	-0.682276
135.H	3.284406	1.564430	-0.705087
136.H	2.773697	3.964635	-0.644229
137.H	-1.788609	6.839560	-0.242043
138.H	3.674346	5.489924	-0.715490
139.H	-1.474569	-2.355469	-0.674510
140.H	-1.296571	-4.054874	-0.350937
141.H	-1.590427	4.354607	-0.218818
142.H	0.792609	5.432164	0.439835
143.H	1.904542	6.797714	0.245181
144.H	-4.031513	3.867160	0.069910
145.H	4.387791	4.070989	0.073628
146.H	-6.512819	1.630568	0.275597
147.H	6.162622	-2.194190	0.557628
148.H	-5.710358	-3.863205	0.567264
149.H	1.028789	-3.800278	0.392195
150.H	-3.580811	5.442093	0.749145
151.H	-7.351543	-0.729996	0.781711
152.H	-3.282543	1.699301	0.607888
153.H	-8.059340	1.586210	1.150704
154.H	5.223753	2.030054	1.088547
155.H	1.185781	6.466409	1.831295
156.H	-3.194115	-3.367874	1.148108
157.H	6.566332	0.126048	1.391534
158.H	2.328135	-2.790366	1.018939
159.H	-7.001552	-3.145989	1.554045
160.H	2.297925	-4.501241	1.419260
161.H	-0.602355	-0.482568	1.151006
162.H	4.917484	-2.416995	1.768476
163.H	-4.349676	2.302624	1.869078
164.H	3.552129	5.715333	1.758162
165.H	0.310893	2.919963	1.575534
166.H	-6.020972	-4.476713	2.197449
167.H	-6.562046	1.973900	2.012748
168.H	5.114368	3.364057	2.220773
169.H	-0.681849	-4.897676	1.957399
170.H	-2.604172	2.180183	2.155661
171.H	-3.560860	-4.176222	2.684966
172.H	6.902777	-1.103574	2.636610

173.H	0.076617	-2.546733	2.247251
174.H	-2.701598	-0.281177	2.123697
175.H	-0.374029	5.148300	2.466222
176.H	-8.508092	-0.157432	2.905899
177.H	-2.811876	-2.568537	2.681447
178.H	-7.441005	-1.555603	3.136867
179.H	6.523018	1.602400	3.215076
180.H	0.562173	-5.474000	3.080085
181.H	3.401772	-5.346317	3.187292
182.H	-5.239125	-2.378345	3.222594
183.H	4.399132	-3.883902	3.119832
184.H	-0.626692	1.417486	3.141089
185.H	1.095922	1.111183	3.400557
186.H	-1.393056	3.892683	3.182806
187.H	-6.954988	0.041099	3.735312
188.H	4.673267	5.635745	3.474166
189.H	-0.762174	-4.448408	3.669795
190.H	5.159569	4.194198	4.390260
191.H	-0.084548	4.611362	4.133562
192.H	4.993667	1.744634	4.118813
193.H	-4.812189	0.906907	4.027395
194.H	5.115856	-1.778820	3.986510
195.H	4.747542	-5.146966	4.321190
196.H	-1.251413	-1.847152	4.065832
197.H	-3.976704	-0.641960	4.263580
198.H	2.289890	5.297416	4.272577
199.H	-3.060037	0.875381	4.293161
200.H	0.233422	2.127014	4.521529
201.H	5.560189	-0.356588	4.961798
202.H	3.164136	0.442232	4.751525
203.H	-0.374940	-0.439794	4.688219
204.H	4.515859	5.605475	5.239280
205.H	1.689353	-5.695868	5.138249
206.H	3.612246	-3.316803	5.485971
207.H	3.272736	2.633020	5.466156
208.H	3.294999	-1.112001	5.550552
209.H	-1.503707	-1.316058	5.739317
210.H	1.624662	3.276465	5.545612
211.H	-0.022537	-3.345915	5.632970
212.H	3.129375	-5.660431	6.169924
213.H	2.941125	4.104879	6.396975
214.H	1.750747	-4.605309	6.535229
215.H	1.321045	-0.777210	6.664126
216.H	1.802919	-2.417402	7.148709
217.H	0.182525	-1.810192	7.540603
218.As	0.075232	0.093176	-0.118745
219.N	-2.478771	-1.274735	-3.497548
220.N	-4.999753	0.097871	-3.042091
221.N	-3.168226	2.161836	-2.127191
222.N	-4.474829	-0.844139	-0.336442
223.N	4.396594	-0.973350	0.270051
224.N	3.318074	2.162224	2.066607

225.N	5.104049	0.027986	2.904879
226.N	2.541613	-1.204099	3.521289
227.Si	-1.734691	-2.815145	-3.967056
228.Si	-2.234089	3.635879	-2.452240
229.Si	4.811481	-1.025353	-1.453920
230.Si	-4.966729	-0.807583	1.363885
231.Si	2.452352	3.644356	2.523725
232.Si	1.716851	-2.682235	4.050010
233.Th	-2.639857	-0.028529	-1.531495
234.Th	2.667376	-0.014224	1.511074

Energy: −1257.11678380 eV

Supplementary Table 4. Final Coordinates and Single Point Energy of 6⁻ After Geometry Optimisation

1.C	-0.156453	0.454003	-7.969220
2.C	-1.148319	-1.551377	-6.809490
3.C	-1.003201	-0.018061	-6.767499
4.C	1.317291	2.898566	-5.864249
5.C	-0.113121	2.589394	-5.374318
6.C	2.151291	-0.498560	-5.259425
7.C	-4.112435	-0.003904	-4.731464
8.C	-3.108391	1.120601	-4.462902
9.C	-0.451636	3.487401	-4.172724
10.C	1.005926	-0.216359	-4.263867
11.C	-4.597963	-2.140930	-3.584233
12.C	0.575069	-1.521201	-3.564076
13.C	-3.244790	-2.811596	-3.340347
14.C	-0.834582	-5.133017	-2.923691
15.C	-5.628500	-0.046138	-2.787453
16.C	-5.287983	4.071649	-2.441123
17.C	3.766895	2.179549	-2.618774
18.C	-4.597055	-5.851471	-2.177087
19.C	6.109458	2.724905	-1.867212
20.C	4.624865	3.131908	-1.770338
21.C	3.447232	-3.870363	-1.739562
22.C	-7.261623	3.111824	-1.200667
23.C	-1.158884	-5.103601	-1.417422
24.C	-5.396506	-0.004974	-1.276823
25.C	-5.790906	3.562692	-1.077822
26.C	3.487502	-1.412255	-1.146501
27.C	-1.212565	-6.546242	-0.873472
28.C	-2.048030	3.379177	-1.036725
29.C	1.860214	5.065125	-1.019915
30.C	-4.237699	-5.108146	-0.872701
31.C	2.979928	-2.804565	-0.726059
32.C	-5.504910	-4.466483	-0.277839
33.C	5.487736	5.820390	-0.034264
34.C	2.098837	3.849214	-0.103395

35.C	-2.938143	3.220036	0.208840
36.C	6.264562	-3.240924	0.550241
37.C	5.087466	4.642401	0.880554
38.C	-3.072739	4.589656	0.903160
39.C	2.169919	-5.938726	1.063053
40.C	5.120725	-4.116016	1.099948
41.C	-2.298555	-3.321239	0.848175
42.C	5.305895	1.292811	1.255362
43.C	1.389546	4.066880	1.245364
44.C	-5.595137	1.928651	1.492322
45.C	-3.228417	-2.196235	1.327802
46.C	2.049956	-4.536975	1.692741
47.C	-2.237744	-4.433090	1.914541
48.C	4.559309	5.193538	2.219543
49.C	5.583870	-4.837114	2.382577
50.C	-6.117832	3.133428	2.301099
51.C	-4.861784	0.943304	2.415472
52.C	5.540476	1.335030	2.766002
53.C	1.897618	-4.665876	3.220574
54.C	3.840208	-1.884359	3.434768
55.C	5.016217	-0.922690	3.607972
56.C	-1.374071	3.095559	3.810008
57.C	-0.143202	-1.610999	3.915669
58.C	-0.814986	-0.255420	4.192837
59.C	2.874768	1.971476	4.464449
60.C	4.077745	1.064298	4.727748
61.C	-2.117540	-0.461138	4.991290
62.C	-0.650676	2.715379	5.111310
63.C	0.189806	3.906505	5.613014
64.C	0.900822	0.639606	6.784769
65.C	1.404966	-0.803804	6.980901
66.C	-0.142337	0.972309	7.873342
67.H	-0.572143	0.061773	-8.915403
68.H	-0.126248	1.549662	-8.055335
69.H	0.883376	0.096856	-7.903455
70.H	-1.596596	-1.881275	-7.764096
71.H	-2.015775	0.404218	-6.924137
72.H	-0.171858	-2.052198	-6.722287
73.H	1.599575	2.322902	-6.756890
74.H	-1.782028	-1.935118	-5.996996
75.H	-0.804780	2.866615	-6.194849
76.H	1.842052	-1.200757	-6.049412
77.H	1.417244	3.969246	-6.116902
78.H	2.521707	0.411530	-5.749512
79.H	-3.635575	-0.740598	-5.391090
80.H	-2.963969	1.688247	-5.401509
81.H	-5.022333	0.369004	-5.245151
82.H	2.058186	2.683335	-5.080044
83.H	3.005193	-0.960394	-4.735508
84.H	-0.236241	4.546735	-4.398794
85.H	-5.019986	-2.413927	-4.573218
86.H	-0.157411	-2.102268	-4.140371

87.H	-2.532160	-2.425431	-4.094121
88.H	-1.508063	3.411450	-3.890685
89.H	-3.574981	1.840580	-3.765164
90.H	4.142682	2.118929	-3.655307
91.H	-3.345015	-3.885455	-3.568829
92.H	-1.621077	-5.643226	-3.503538
93.H	1.442978	-2.176881	-3.390215
94.H	1.404604	0.447814	-3.475290
95.H	0.137522	3.215126	-3.284327
96.H	-5.721554	0.987225	-3.147831
97.H	-5.199439	3.254039	-3.174321
98.H	-0.716957	-4.122604	-3.334319
99.H	-6.566082	-0.579545	-3.048209
100.H	-5.986280	4.814914	-2.867989
101.H	0.106193	-5.680016	-3.110369
102.H	6.461032	2.771635	-2.913643
103.H	-5.297316	-2.492386	-2.813742
104.H	-5.042515	-5.173633	-2.921932
105.H	3.093032	-3.617049	-2.753150
106.H	-3.726707	-6.332655	-2.647014
107.H	0.183033	-1.322741	-2.546896
108.H	2.715661	2.496572	-2.663188
109.H	-4.302365	4.550642	-2.369640
110.H	4.535358	4.146149	-2.207768
111.H	3.778265	1.162706	-2.202111
112.H	-7.889471	3.931773	-1.594129
113.H	-5.344582	-6.642032	-1.981315
114.H	3.483737	-1.309295	-2.243955
115.H	-7.373198	2.265652	-1.897158
116.H	2.292113	4.927173	-2.021075
117.H	4.545487	-3.938076	-1.791553
118.H	6.260325	1.688915	-1.527150
119.H	3.068514	-4.874665	-1.505678
120.H	-2.385688	4.212126	-1.670866
121.H	-2.008687	-7.132546	-1.361121
122.H	6.771853	3.370487	-1.270164
123.H	-2.019029	2.496667	-1.696296
124.H	-0.260884	-7.071710	-1.066462
125.H	4.606059	6.373951	-0.396118
126.H	-6.323744	0.353478	-0.794154
127.H	-5.845476	-3.602703	-0.871603
128.H	6.053692	5.493269	-0.918526
129.H	4.506554	-1.188680	-0.801454
130.H	-5.271573	-1.044495	-0.917765
131.H	-0.307259	-4.596916	-0.923698
132.H	0.781216	5.248514	-1.152032
133.H	2.293015	5.984190	-0.592363
134.H	1.876210	-2.756971	-0.776288
135.H	-1.003767	3.571100	-0.753078
136.H	-5.787677	4.436137	-0.395088
137.H	-7.692098	2.803826	-0.236802
138.H	2.796788	-0.619987	-0.798387

139.H	6.033295	-2.821744	-0.439047
140.H	-6.339384	-5.191901	-0.251589
141.H	1.606776	2.972566	-0.573751
142.H	-3.908477	-5.887332	-0.154566
143.H	2.136562	-5.912390	-0.035060
144.H	-1.393137	-6.589929	0.209655
145.H	6.119631	6.543698	0.513808
146.H	7.197647	-3.825484	0.451310
147.H	4.944223	-4.912936	0.349254
148.H	-3.615423	5.310778	0.269541
149.H	-5.349108	-4.112239	0.750477
150.H	6.116658	1.865364	0.771684
151.H	-3.330338	-1.365935	0.610578
152.H	6.019505	4.087923	1.107531
153.H	5.443404	0.251357	0.907911
154.H	-2.398278	2.537248	0.892916
155.H	-2.078942	5.022482	1.106945
156.H	-1.288724	-2.869820	0.767029
157.H	6.483967	-2.393483	1.218833
158.H	3.107969	-6.439265	1.355000
159.H	1.340373	-6.588209	1.394186
160.H	-6.489619	1.397515	1.110098
161.H	0.303383	4.163377	1.098866
162.H	1.109968	-4.084970	1.319994
163.H	-6.656641	3.866042	1.681585
164.H	-4.248470	-2.555172	1.528775
165.H	1.737362	4.980530	1.748758
166.H	-3.601923	4.526553	1.863008
167.H	6.471625	-5.463421	2.176811
168.H	-1.434036	-5.154721	1.715525
169.H	-3.184187	-4.994463	1.979345
170.H	3.713746	5.880135	2.059465
171.H	-4.556853	0.037813	1.878969
172.H	1.533840	3.241293	1.961137
173.H	5.342106	5.765388	2.749627
174.H	-2.844839	-1.743514	2.253756
175.H	4.808809	-5.495718	2.800570
176.H	-6.810217	2.799623	3.094685
177.H	4.208955	4.400975	2.896237
178.H	-5.295448	3.666755	2.802360
179.H	5.749131	-1.130970	2.816888
180.H	5.876045	-4.126815	3.172361
181.H	6.575877	1.036590	3.031609
182.H	-2.047181	-4.007736	2.913699
183.H	-5.500800	0.637766	3.262983
184.H	-3.951047	1.391335	2.840180
185.H	5.395099	2.369665	3.104576
186.H	-0.666881	3.226932	2.979276
187.H	1.027524	-5.297646	3.470624
188.H	-0.788540	-2.250902	3.296899
189.H	2.779345	-5.136444	3.684430
190.H	-1.084376	0.164989	3.202108

191.H	4.189465	-2.901771	3.675770
192.H	1.744936	-3.692363	3.701703
193.H	-2.093911	2.326762	3.498348
194.H	0.809060	-1.528827	3.366273
195.H	3.190277	2.777769	3.777040
196.H	-1.928858	4.042689	3.926665
197.H	3.087908	-1.650603	4.212692
198.H	5.523092	-1.065283	4.584793
199.H	-2.809922	-1.130033	4.453410
200.H	0.959615	4.183498	4.876937
201.H	0.079011	-2.156254	4.844304
202.H	4.898846	1.606990	5.240299
203.H	-2.652153	0.482882	5.170311
204.H	3.753574	0.249039	5.388569
205.H	2.623926	2.485035	5.408987
206.H	-0.444064	4.797822	5.766034
207.H	-1.426617	2.513860	5.874989
208.H	-1.920384	-0.923216	5.972331
209.H	0.699584	3.698784	6.566512
210.H	2.130864	-1.106909	6.212223
211.H	0.572523	-1.523388	6.941888
212.H	1.764765	1.308037	6.967383
213.H	-1.062007	0.378312	7.751429
214.H	-0.433657	2.032297	7.865893
215.H	1.890138	-0.925357	7.966364
216.H	0.256588	0.747043	8.879338
217.As	-0.007952	0.110051	0.039948
218.N	-1.845211	0.581077	-3.909454
219.N	-4.467175	-0.671422	-3.458294
220.N	-2.757793	-2.551430	-1.967062
221.N	-4.212481	0.819912	-0.944328
222.N	3.956497	1.795804	0.914633
223.N	3.258687	-1.777530	2.076280
224.N	4.554497	0.474941	3.456794
225.N	1.743258	1.203731	3.900211
226.Si	-0.490758	0.713626	-5.027806
227.Si	-2.657666	-3.964130	-0.925368
228.Si	-4.603962	2.332599	-0.130405
229.Si	3.937656	3.312987	0.027521
230.Si	3.373200	-3.244722	1.110150
231.Si	0.343747	1.064196	4.959951
232.Th	-2.221109	-0.253314	-1.714806
233.Th	2.261829	0.349277	1.738660

Energy: −1255.28500919 eV

Supplementary Methods

General Experimental Details

All manipulations were carried out using standard Schlenk techniques, or an MBraun UniLab glovebox, under an atmosphere of dry nitrogen. Solvents were dried by passage through activated alumina towers and degassed before use. All solvents were stored over potassium mirrors except for ethers, which were stored over activated 4 Å sieves. Deuterated solvent was distilled from potassium, degassed by three freeze-pump-thaw cycles and stored under nitrogen. The compounds [Th(Tren^{TIPS})(DME)][BPh_4] (**1**), [Th{N($CH_2CH_2NSiPr^i_3$)$_2$($CH_2CH_2NSiPr^i_2$C[H]MeCH_2)}] (**4**), benzyl potassium, and $KAsH_2$ were prepared as described previously.[1-4] 15-crown-5 ether was dissolved in ether, stored over activated 4 Å sieves for 18 hours, then decanted and solvent removed *in vacuo* prior to use.

^{1}H, ^{13}C{^{1}H}, and ^{29}Si NMR spectra were recorded on a Bruker 400 spectrometer operating at 400.1, 100.6, and 79.5 MHz, respectively; chemical shifts are quoted in ppm and are relative to TMS (^{1}H, ^{13}C. ^{29}Si). FTIR spectra were recorded on a Bruker Alpha spectrometer with Platinum-ATR module. Elemental microanalyses were carried out by Mr Martin Jennings and Mrs Anne Davies at The University of Manchester School of Chemistry Microanalysis service. Crystal structures are deposited with the Cambridge Structural Database, CCDC numbers 1518196-1518199.

General Computational Details

Restricted geometry optimisations were performed for the full models of **2**, **3**, and **5** and the full anion component of **6** (**6**$^{-}$) using coordinates derived from the X-ray crystal structures. No constraints were imposed on the structures during the geometry optimisations. The calculations were performed using the Amsterdam Density Functional (ADF) suite version 2012.01.[5,6] The DFT geometry optimisations employed Slater type orbital (STO) triple-ζ-plus polarisation all-electron

basis sets (from the ZORA/TZP database of the ADF suite). Scalar relativistic approaches were used within the ZORA Hamiltonian for the inclusion of relativistic effects and the local density approximation (LDA) with the correlation potential due to Vosko *et al*[7] was used in all of the calculations. Gradient corrections were performed using the functionals of Becke[8] and Perdew.[9] MOLEKEL[10] was used to prepare the three-dimensional plots of the electron density. Natural Bond Order (NBO) analyses were carried out with NBO 5.0.[11] The Atoms in Molecules analysis[12,13] was carried out with Xaim-1.0.[14]

Supplementary References

1. Wildman, E. P., Balázs, G., Wooles, A. J., Scheer, M. & Liddle, S. T. Thorium-Phosphorus Triamidoamine Complexes Containing Th-P Single- and Multiple-Bond Interactions. *Nat. Commun.* **7**:12884 (2016).
2. Gardner, B. M. *et al.* The role of 5f-orbital participation in unexpected inversion of the σ-bond metathesis reactivity trend of triamidoamine thorium(IV) and uranium(IV) alkyls. *Chem. Sci.* **5**, 2489-2497 (2014).
3. Bailey, P. J. *et al.* The first structural characterisation of a group 2 metal alkylperoxide complex: comments on the cleavage of dioxygen by magnesium alkyl complexes. *Chem. Eur. J.* **9**, 4820-4828 (2003).
4. Adapted from Johnson, W. C. & Pechukas, A. Hydrogen compounds of arsenic. II. Sodium and potassium dihydrogen arsenides. *J. Am. Chem. Soc.* **59**, 2068-2071 (1937).
5. Fonseca-Guerra, C., Snijders, J. G., Velde, G. Te & Baerends, E. J. Towards an order-N DFT method. *Theor. Chem. Acc.* **99**, 391-403 (1998).
6. Velde, G. *et al*. Chemistry with ADF. *J. Comput. Chem.* **22**, 931-967 (2001).
7. Vosko, S. H., Wilk, L. & Nusair, M. Accurate spin-dependent electron liquid correlation energies for local spin density calculations: A critical analysis. *Can. J. Phys.* **58**, 1200-1211 (1980).

8. Becke, A. D. Density-functional exchange-energy approximation with correct asymptotic behavior. *Phys. Rev. A* **38**, 3098-3100 (1988).

9. Perdew, J. P. Density-functional approximation for the correlation energy of the inhomogeneous electron gas. *Phys. Rev. B* **33**, 8822-8824 (1986).

10. Portmann, S. & Luthi, H. P. MOLEKEL: An interactive molecular graphics tool. *Chimia* **54**, 766-770 (2000).

11. NBO 5.0: Glendening, E. D., Badenhoop, J. K., Reed, A. E., Carpenter, J. E., Bohmann, J. A., Morales, C. M., Weinhold F. Theoretical Chemistry Institute, University of Wisconsin, Madison, WI, 2001; http://www.chem.wisc.edu/~nbo5.

12. Bader, R. F. W. *Atoms in Molecules: A Quantum Theory*, Oxford University Press, New York, 1990.

13. Bader, R. F. W. A bond path: A universal indicator of bonded interactions. *J. Phys. Chem. A* **102**, 7314-7323 (1998).

14. http://www.quimica.urv.es/XAIM.
